# Altered peripheral taste function in a mouse model of inflammatory bowel disease

**DOI:** 10.1038/s41598-023-46244-3

**Published:** 2023-11-02

**Authors:** Guangkuo Dong, Khaylie Boothe, Lianying He, Yang Shi, Lynnette Phillips McCluskey

**Affiliations:** 1 Department of Neuroscience and Regenerative Medicine, Medical College of Georgia, Augusta University, 1120 15th Street/CA-3016, Augusta, GA 30912 USA; 2 Division of Biostatistics and Data Science, Department of Population Health Sciences, Medical College of Georgia, Augusta University, Augusta, GA 30912 USA; 3 Present Address: Department of Cell Biology and Physiology, University of North Carolina at Chapel Hill, Chapel Hill, NC 27599 USA

**Keywords:** Neuroscience, Gustatory system, Taste receptors

## Abstract

Increased sugar intake and taste dysfunction have been reported in patients with inflammatory bowel disease (IBD), a chronic disorder characterized by diarrhea, pain, weight loss and fatigue. It was previously unknown whether taste function changes in mouse models of IBD. Mice consumed dextran sodium sulfate (DSS) during three 7-day cycles to induce chronic colitis. DSS-treated mice displayed signs of disease, including significant weight loss, diarrhea, loss of colon architecture, and inflammation of the colon. After the last DSS cycle we assessed taste function by recording electrophysiological responses from the chorda tympani (CT) nerve, which transmits activity from lingual taste buds to the brain. DSS treatment significantly reduced neural taste responses to natural and artificial sweeteners. Responses to carbohydrate, salt, sour or bitter tastants were unaffected in mice with colitis, but umami responses were modestly elevated. DSS treatment modulated the expression of receptor subunits that transduce sweet and umami stimuli in oral taste buds as a substrate for functional changes. Dysregulated systemic cytokine responses or dysbiosis that occurs during chronic colitis may be upstream from changes in oral taste buds. We demonstrate for the first time that colitis alters taste input to the brain, which could exacerbate malnutrition in IBD patients.

## Introduction

Inflammatory bowel disease (IBD), a term that includes Crohn’s disease and ulcerative colitis, causes diarrhea, pain, weight loss, fatigue, and potentially lethal complications from intestinal inflammation^1, 2^. While symptoms are similar in these two conditions, inflammation is continuous and limited to the large intestine in ulcerative colitis, but interspersed with healthy tissue throughout the gastrointestinal (GI) tract in Crohn’s disease^1^. IBD is a chronic condition, with symptoms typically cycling between flare-ups and remission. More than 85% of IBD patients are malnourished due to changes in food intake and absorption^3–5^.

The taste system plays a critical role in food selection and nutrition^6^, so gustatory dysfunction could exacerbate changes in body weight. The increased sweet consumption reported in Crohn’s patients could also result from changes in taste function^7–9^. Multiple studies have shown decreased sensitivity to one or more taste qualities in at least a subset of IBD patients^7–12^, though taste was unaltered in other reports^12, 13^. In a recent study, sensitivity to sweet, salty, bitter, and umami stimuli was reduced and sour sensitivity increased in IBD patients^11^. Well-established mouse models of IBD have been critical in identifying inflammatory mechanisms in colitis^2, 14–16^, but have not been used to study the functional underpinnings of psychophysical taste changes.

The chorda tympani (CT) nerve transmits signals encoding taste quality from anterior lingual taste buds to the brain. Taste buds are composed of a heterogeneous population of receptor cells, including type I glial-like cells, type II cells responsive to sweet, bitter, and umami stimuli, and type III cells responsive to sour stimuli^17^. G-protein coupled receptors expressed by type II taste cells transduce complex taste stimuli. These include T1R2 + T1R3 heterodimers that bind sweet tastants, T1R1 + T1R3 receptors for umami stimuli, and T2Rs that transduce bitter stimuli^18^. Canonical taste receptors and downstream signaling molecules are also expressed in extraoral tissues including the gut^18–20^.

Communication between the gut and oral taste buds is well established. For example, peptides that regulate metabolism signal oral taste receptor cells in addition to hypothalamic neurons^21–23^. GI inflammation can also affect the taste system. Peripheral taste function is dysregulated by ingestion or enteral delivery of the inflammatory stimulus, lipopolysaccharide (LPS), derived from gram-negative bacteria^24, 25^. LPS ingested during a single overnight period transiently reduced CT nerve responses to sucrose in mice^25^. Both acute and chronic LPS gavage, which bypasses oral taste buds, altered taste more extensively. Behavioral and neurophysiological responses to natural and artificial sweeteners and NaCl were reduced in mice gavaged with LPS once or weekly^24^. Though chronic LPS gavage increased neutrophils in the small intestine and colon, mice did not display sickness behavior, weight loss or gut leakiness. Thus, peripheral taste sensitivity to sweet and salt stimuli was impaired by relatively mild inflammation localized to the GI tract^24^. These studies, together with reports of altered taste in IBD patients^7–12^, provide a rationale for testing taste function and potential mechanisms underlying changes in mice with colitis.

In the most widely used mouse model of relapsing IBD, dextran sodium sulfate (DSS)-treated drinking water is cycled on and off to allow intermittent recovery periods. While the mechanisms underlying DSS-induced colitis are incompletely understood, this treatment breaks down the epithelium in the colon, causing bacterial entry to the lumen and inflammation^14, 16, 26, 27^. The DSS-induced colitis model is straightforward, reproducible, targets the distal colon, and has provided insight to innate immune responses and the influence of the microbiome in IBD^14, 16, 26, 27^. Intriguingly, DSS-induced colitis is more severe in mice lacking α-gustducin, a G-protein subunit expressed by a subset of intestinal epithelial cells^28, 29^. This supports growing evidence for the immunoregulatory role of taste signaling molecules in in tissues outside of the oral cavity^20^. Taste transduction components in oral taste buds may also be targets of inflammatory changes in the GI tract^30^.

We hypothesized that DSS-induced colitis alters peripheral taste function. To test this, we recorded electrophysiological taste responses from the CT nerve of mice given three cycles of DSS, which elicited inflammation of the colon and symptomatic disease as expected. DSS treatment had striking effects on sweet taste function, reducing taste responses to all natural and artificial sweet stimuli tested. CT responses to the umami stimulus, monosodium glutamate (MSG), were modestly increased. *Tas1r2* was downregulated and *Tas1r1* expression elevated in lingual taste buds of DSS-treated compared to control mice, as a molecular substrate for neural changes in sweet and umami responses, respectively. These results demonstrate major changes in sweet taste sensing in a mouse model of IBD, and provide a basis for further mechanistic studies linking gut inflammation and the regulation of taste transduction genes in lingual taste buds.

## Results

### Chronic DSS treatment induces body weight loss, diarrhea, and inflammation of the colon

We initially provided male and female mice with 3% DSS in drinking water or water alone from day 1–7, removed DSS, then recorded CT nerve responses 21–25 days after the start of the experiment. This treatment did not alter neural responses to any taste quality (Fig. S1a-c). DSS-treated males lost significantly more weight than control males on day 2, 3, and 5–8 (Fig. S1d; statistical test results in Table S1), while body weights were similar in treated and control females (*p* < 0.05).

**Figure 1:**
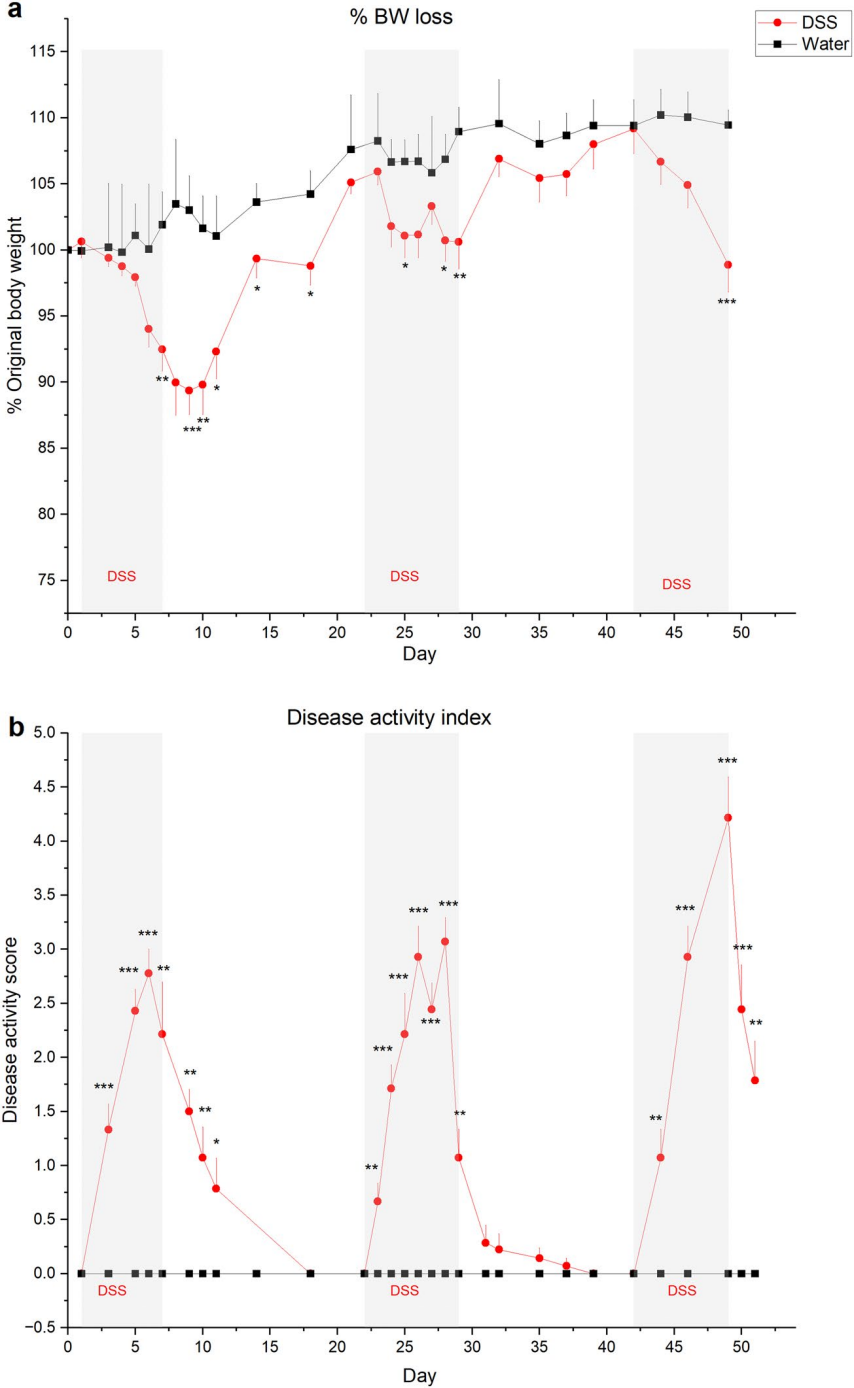
7-day cycles of 2% DSS in drinking water induce colitis. (**a)** Mean (+ SEM) body weight expressed as a percentage of original body weight over time. Mice lost significantly more body weight at several time points during or after DSS treatment (*n* = 13) compared to water-treated controls (*n* = 5). Grey shaded blocks indicate DSS treatment days. (**b**) Mean Disease Activity Index scores (+ SEM) were significantly elevated on most days in DSS-treated mice compared to controls. Individual scores representing the magnitude of body weight loss, blood in stool, and diarrhea are summed to calculate the Disease Activity score as described in Methods. *p < 0.05; **p < 0.001, ***p < 0.0001. Actual *p* values are listed in Table 1.

**Figure 2:**
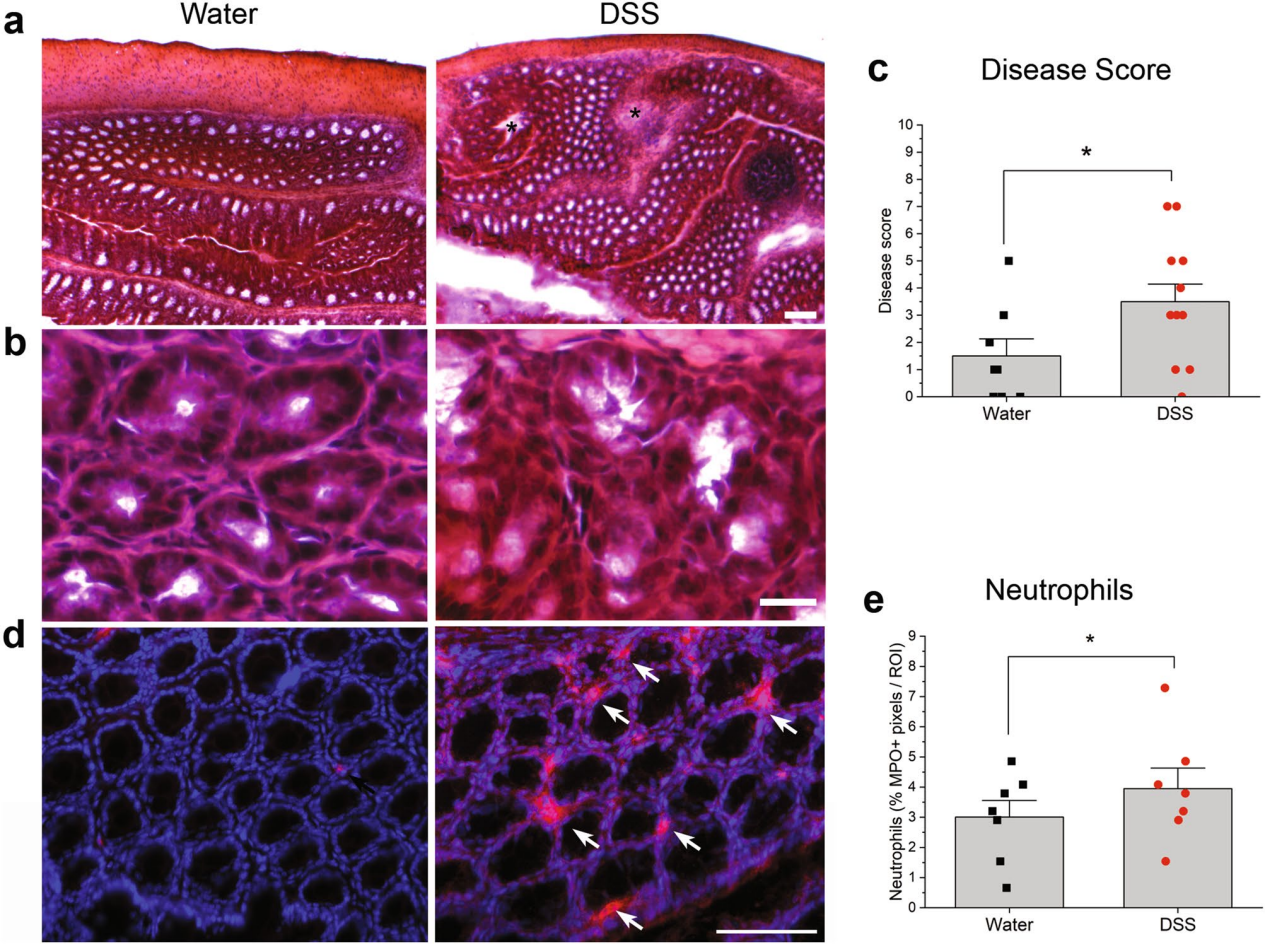
DSS treatment elicited inflammation and loss of tissue structure in the colon. (**a, b**) Hematoxylin and eosin staining reveals the loss of tissue architecture in the colon of mice treated with DSS. Black stars indicate severe tissue damage and inflammation. (**c**) Disease scores reflecting inflammation and tissue breakdown (see Methods) were significantly higher in mice drinking DSS (*n* = 6) compared to controls (*n* = 4). (**d**) Myeloperoxidase (MPO)-expressing neutrophils (red) are more prominent in the colon of DSS-treated mice. Blue nuclei are stained with DAPI. (**e**) Neutrophil density was significantly higher in DSS-treated mice (*n* = 4) compared to controls (*n* = 4). *p < 0.05. Bar in a = 100 µm; b = 20 µm; c = 75 µm.

Since males but not females exhibited significant weight loss in acute experiments as previously reported^31^, we used males to test whether chronic DSS alters peripheral taste responsiveness. DSS was provided in three cycles on days 1–7, 22–29 and 42–49. This treatment induces chronic, relapsing and remitting colitis mimicking IBD^32, 33^. As shown in Fig. 1a, mice lost significantly more body weight on days during and immediately following 2% DSS treatment cycles compared to controls. Actual *p* values are shown in Table 1. Recovery of body weight between DSS treatment mimicked remissions characteristic of IBD^26^. The disease activity index was significantly elevated during and after DSS treatment, compared with control scores that remained at baseline throughout the study (Fig. 1b; Table 1). This measure sums individual scores for body weight loss, stool consistency, and blood in stool^34^. Hemotoxylin and eosin stained sections of colon were also scored according to standard methods^32, 34^. Colons from DSS-treated mice displayed inflammatory cell infiltrates, loss of tissue architecture (Fig. 2a) and significantly elevated disease scores (Fig. 2b) compared to water-treated controls (*p* = 0.024)^32, 34^. Myeloperoxidase + neutrophils were also more abundant in colons from DSS-treated mice (Fig. 2c,d) (*p* = 0.029) Together, these results verify that DSS treatment induced inflammation, colon damage, diarrhea, and body weight loss typical of the model^14, 26, 29, 34^.

**Table 1:** Statistical test results for percent body weight loss and disease activity index (DAI) analyses DSS-treated versus water controls.

Day^a^	*t* statistic BW loss	*p* value BW loss	*W* statistic (rank-sum) DIA score	*p* value DIA score
0	N/A	N/A	N/A	N/A
**1**	0.5519	0.6057	ND^b^	ND
**3**	− 0.1658	0.8785	68	0.0009***
**4**	− 0.2049	0.8503	ND	ND
**5**	− 1.2720	0.2388	112	0.0001***
**6**	− 1.1891	0.3093	72	0.0002***
**7**	− 3.1801	0.0073**	92	0.0061**
8	− 2.4767	0.0601	ND	ND
9	− 4.3174	0.0007***	112	0.0001***
10	− 3.5489	0.0024**	92	0.0061**
11	− 2.3854	0.0325*	88	0.0108*
14	− 2.1199	0.0477*	60	0.5083
18	− 2.3777	0.0305*	56	c
21	− 0.5973	0.5894	ND	ND
**22**	ND	ND	56	c
**23**	− 0.6273	0.5692	60	0.0064**
**24**	− 2.0859	0.0524	108	0.0002***
**25**	− 2.3947	0.0276*	108	0.0002***
**26**	− 2.0798	0.0537	112	0.0001***
**27**	− 0.5641	0.6058	72	0.0002***
**28**	− 2.4891	0.0242*	112	0.0001***
**29**	− 3.0686	0.0063**	92	0.0062**
31	ND	ND	68	0.1882
32	− 0.7481	0.4961	44	0.1967
35	− 1.0310	0.3157	64	0.3044
37	− 1.2455	0.2290	60	0.5083
39	− 0.5263	0.6052	56	c
**42**	− 0.0866	0.9320	56	c
**44**	− 1.3591	0.1925	92	0.0062**
**46**	− 2.0058	0.0611	112	0.0001***
**49**	− 4.4971	0.0003***	112	0.0001***
50	ND	ND	68	0.0009***
51	− 2.3491	0.0445*	100	0.0014**

^a^Mice treated with DSS on days in bold font.

^b^Not determined; small sample size (n < 2) or no data in at least one group (ND).

^c^All data are 0, so the *p*-value cannot be calculated. Conclusion is no difference between the two groups.

### DSS treatment alters neural taste responses

**Figure 3:**
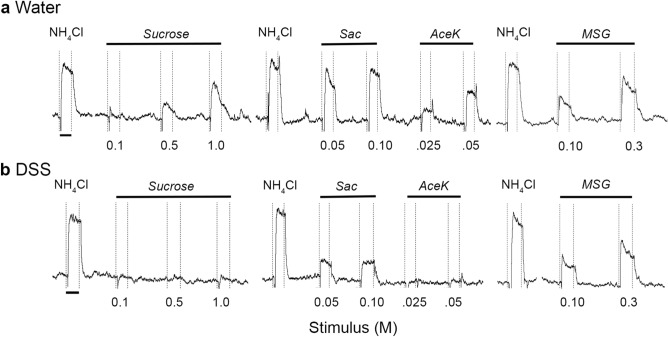
Chorda tympani nerve responses to taste stimuli. Representative responses from (**a**) water-treated control or (**b**) DSS-treated mouse. Responses to 0.5 M NH_4_Cl were robust in both groups, and were used as a standard stimulus to check the stability of the preparation. Responses to sucrose and artificial sweeteners are suppressed in the DSS-treated mouse. Dotted vertical lines indicate stimulus onset and rinse. Black bars under the first 0.5 M NH_4_Cl response indicate 20 s.

**Figure 4:**
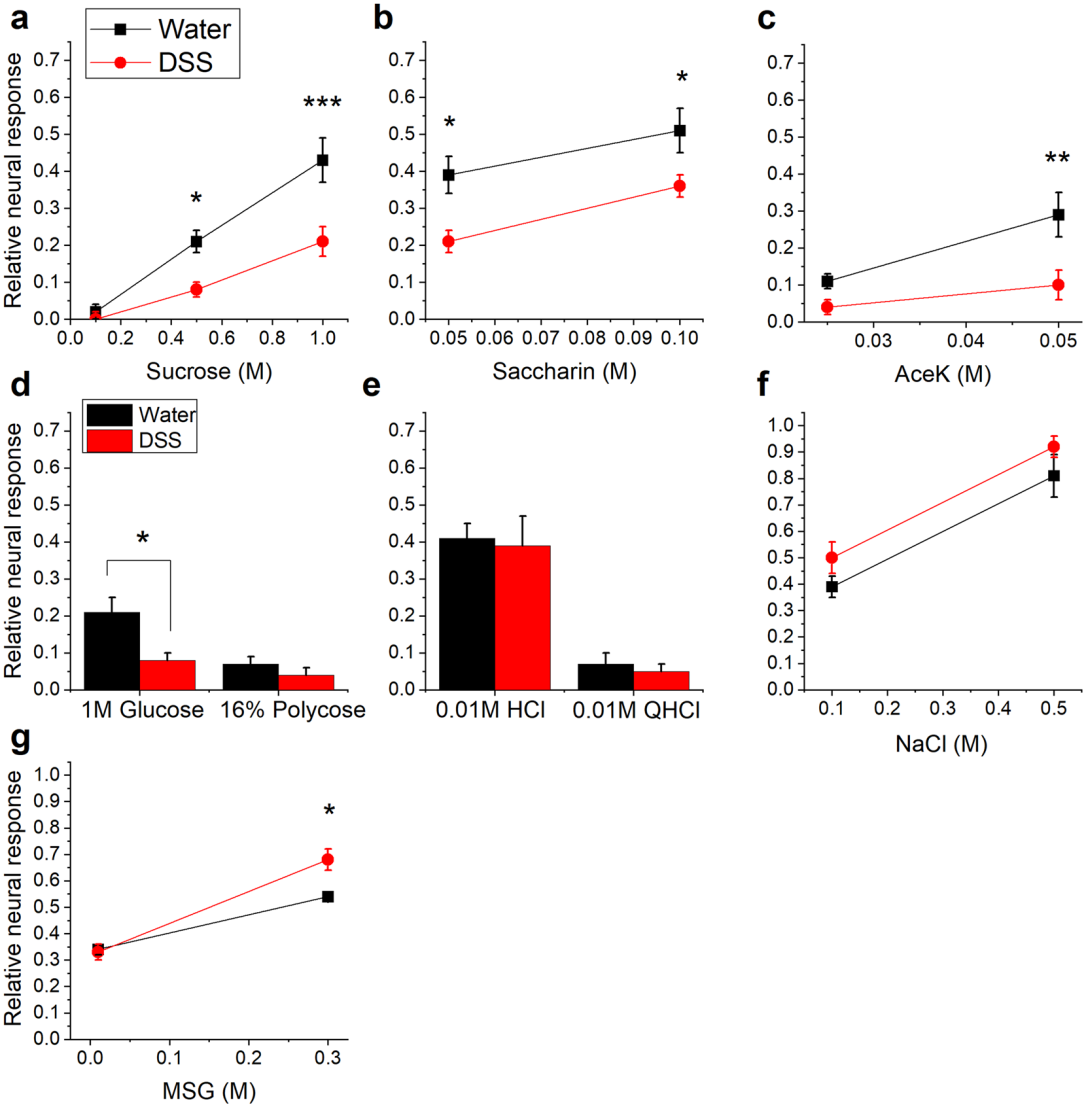
Taste responses to natural and artificial sweeteners are significantly reduced in mice with DSS-induced colitis. Mean neural taste responses (+ SEM) to taste stimuli, expressed relative to 0.5 M NH4Cl responses, and were recorded 56–61 days after the start of the experiment. Three cycles of DSS significantly inhibited responses to (**a**) 0.5 M and 1.0 M sucrose, (**b**) both concentrations of saccharin, (**c**) the higher concentration of aceK, and (**d**) glucose. Neural response magnitudes to (**d**) the starch, polycose, (**e**) HCl, QHCl, and (**f**) NaCl were similar in DSS-treated (*n* = 15) and control (*n* = 8) groups. (**g**) There was a slight but significant increase in responses to the umami stimulus, 0.3 M MSG, n mice with colitis. *p < 0.05; **p < 0.01; ***p < 0.0001.

Representative response traces recorded from the CT nerve are shown in Fig. 3. Neural responses to the standard stimulus, 0.5 M NH_4_Cl, were robust in groups given DSS or maintained on water (Fig. 3a,b). In contrast, responses to natural and artificial sweeteners were suppressed in DSS-treated mice (Fig. 3a,b). Analyses of mean relative response magnitudes demonstrate that colitis had the greatest influence on sweet taste responsiveness (Fig. 4). Sucrose responses were significantly affected by treatment [*F*(1, 19) = 12.78; *p* = 0.002], stimulus concentration, as expected [*F*(2, 38) = 54.27; *p* < 0.0001], and their interaction [*F*(2, 38) = 5.386; *p* = 0.0089]. Specifically, DSS treatment reduced CT responses to 0.5 M (*p* = 0.0282) and 1.0 M (*p* = 0.0001) sucrose (Fig. 4a). There were also significant main effects of treatment [*F*(1, 16) = 7.038; *p* = 0.0174] and stimulus concentration [*F*(1, 16) = 103.4; *p* < 0.001] as chronic DSS reduced responses to 0.05 M and 0.1 M saccharin (Fig. 4b). Significant main effects of treatment [*F*(1, 16) = 8.588; *p* = 0.0098] and stimulus concentration [*F*(1, 16) = 10.75; *p* = 0.004] were similarly observed in decreased responsivity to the higher concentration of the artificial sweetener, acesulfame potassium (aceK; *p* = 0.0044) (Fig. 4c). Neural responses to 1.0 M glucose were also reduced (*p* = 0.0054) in DSS-treated compared to control mice (Fig. 4d). In contrast, neural response magnitudes to the polysaccharide starch, polycose (Fig. 4d), HCl, quinine (QHCl)(Fig. 4e) and NaCl (Fig. 4f) were similar in the two groups. There were significant main effects of stimulus concentration [*F*(1,7) = 260.1; *p* < 0.0001] and the interaction with DSS treatment [*F*(1, 7) = 18.85; *p* = 0.0034], but not treatment alone on MSG responses. Post-tests demonstrated increased responsiveness to 0.3 M MSG in the DSS group (Fig. 4g). Together, neurophysiological results demonstrate that chronic colitis inhibits responses to at least one concentration of each sweet-tasting stimulus tested while modestly elevating umami responses.

### DSS-induced colitis alters lingual taste bud expression of taste transduction genes

**Figure 5:**
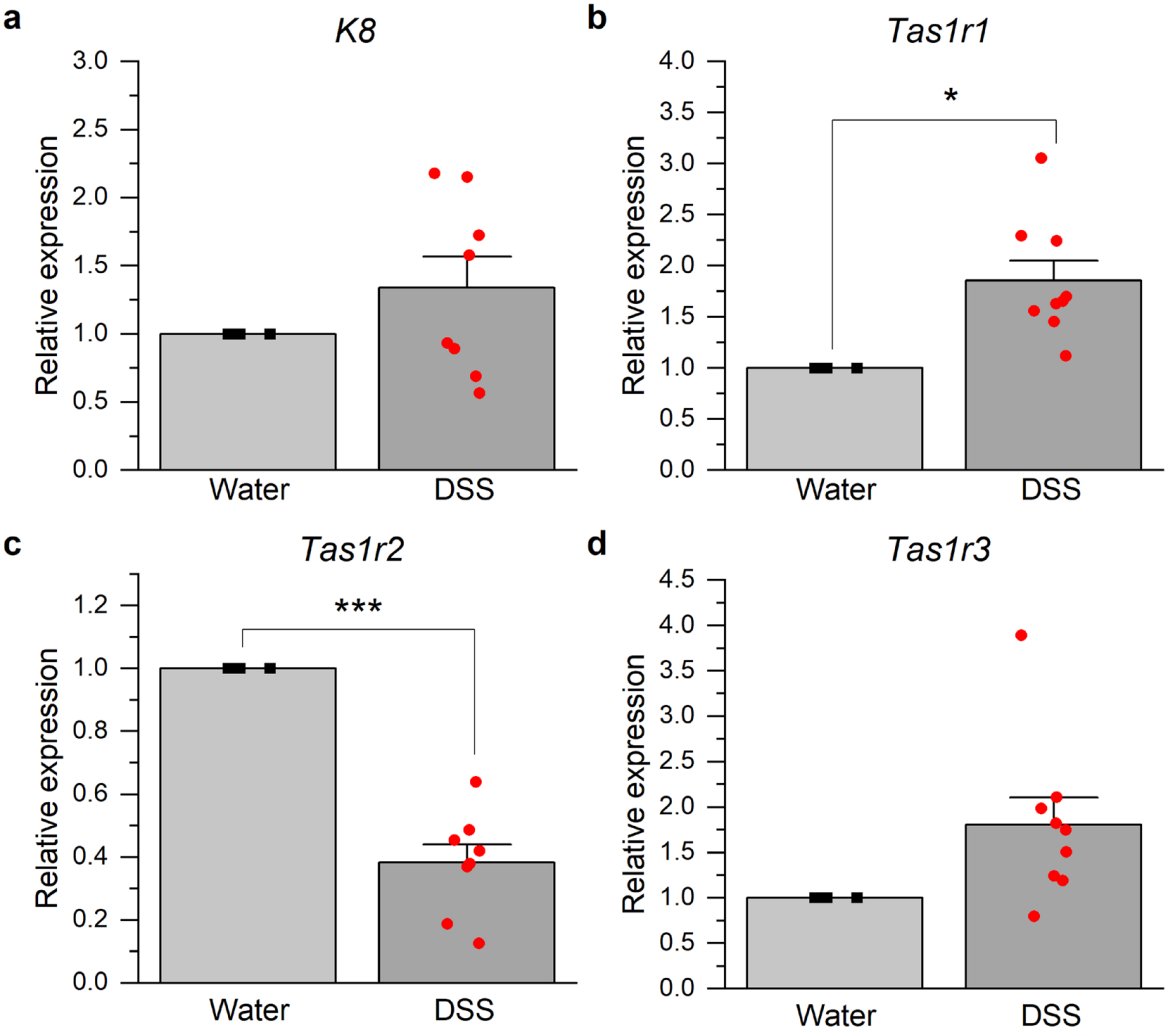
DSS-induced colitis modulates the expression of lingual sweet and umami taste receptor genes. Lingual mRNA expression in DSS-treated mice (*n* = 9) was expressed relative to values for water controls (*n* = 4). (**a)** The expression of the taste receptor cell marker gene, keratin *(K)8*, was similar between groups. (**b**) The umami taste receptor subunit, *Tas1r1*, was significantly increased in mice with colitis compared to controls. (**c**) Expression of the sweet taste receptor subunit, *Tas1r2*, was significantly reduced by DSS treatment. (**d**) Expression levels of *Tas1r3*, which forms heteromeric umami receptors with *Tas1r1* and sweet receptors with *Tas1r2*, were similar between groups. *p < 0.05; ***p < 0.0001.

We next tested whether colitis modulates alters taste function by modulating taste transduction genes. We used quantitative real-time PCR (qPCR) to analyze the expression of keratin (K)8, a marker of mature taste receptor cells, and taste receptor genes for sweet and umami stimuli (Fig. 5). *K8* expression was similar between groups, indicating that changes in neural taste responses in mice with colitis are not mediated by taste receptor cell gain or loss (Fig. 5a). Comparable CT responses to polycose, salty, acid and bitter stimuli between groups support that conclusion (Fig. 4d,f). However, the umami receptor subunit, *Tas1r1*, was significantly upregulated in DSS-treated mice compared to controls (Fig. 5b) (*p* = 0.0149), in parallel with enhanced neural responses to MSG (Fig. 4g). Expression of the sweet taste subunit, *Tas1r2*, was significantly reduced by DSS (Fig. 5c) (*p* < 0.0001) along with reduced neural responses to sweet stimuli (Fig. 4a–d). Expression of *Tas1r3*, which forms a sweet receptor heterodimer with *Tas1r2* and a umami receptor with *Tas1r1*, was not significantly different between groups. These results indicate that colitis alters the expression of sweet and umami taste transduction genes as a substrate for neural changes.

### Inflammation in the anterior tongue

**Figure 6:**
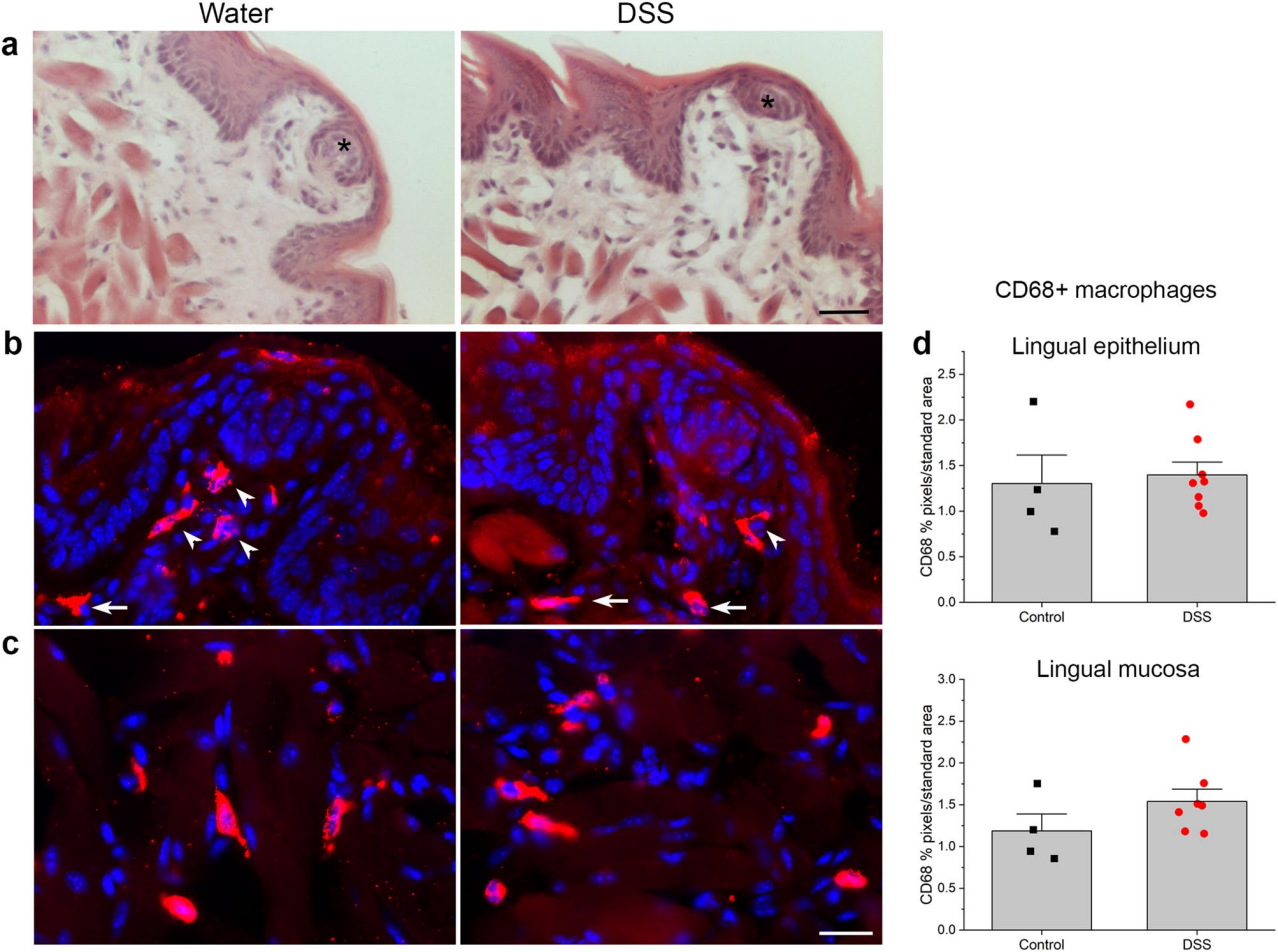
Chronic DSS treatment did not induce lingual inflammation or epithelial breakdown. (**a**) Hematoxylin and eosin stained sections of anterior tongue from control DSS-treated mice demonstrate similar, intact taste buds (black asterisks), surrounding epithelium, and lamina propria without significant inflammatory infiltrates. CD68 + macrophages are similarly dense in (**b**) fungiform papillae, lamina propria and (**c**) underlying dorsal mucosa of the anterior tongue from both groups. (**d**) CD68 + pixels in a standard-sized area of interest from the two tissue compartments were similar between treatment groups (*p* > 0.05). White arrowheads in B indicate macrophages within fungiform papillae, and arrows in the lamina propria. Bar in (**a**) = 30 µm; (**c**) = 20 µm.

We examined tongue sections stained with hematoxylin and eosin to determine if DSS treatment compromised the integrity of the lingual epithelium and elicited inflammation in the tongue (Fig. 6a) similar to colon (Fig. 2a,b). However, the lingual epithelium, fungiform papillae and taste buds were intact and appeared similar in DSS-treated and control groups. We also tested whether inflammation near taste buds could be responsible for changes in neural responses and receptor genes for sweet and umami stimuli. As shown in Fig. 6b–d, however, macrophage density in the epithelium, lamina propria and dorsal mucosa was similar between groups. Thus, while DSS drinking damaged the colon as expected, the lingual epithelium was intact indicating that oral inflammation was not the proximate cause of taste changes.

## Discussion

We demonstrate, for the first time that we are aware of, that taste function is altered in an animal model of IBD. Chronic colitis reduced sweet taste transmission to the brain, in parallel with the downregulation of the *Tas1r2* sweet receptor subunit in oral taste receptor cells. DSS treatment suppressed neural responses to a wide range of natural and artificial sweeteners. Only the two lowest concentrations of sucrose and aceK were unaffected by DSS (Fig. 4a,c), likely because responses were near zero creating a “floor effect”. By the same reasoning, however, we cannot rule out potential effects of colitis on neural responses to the bitter stimulus, QHCl, and the starch, polycose, which is transduced independently of *Tas1r*^35, 36^. The CT nerve responds minimally to these stimuli^24, 25, 37–40^. Chronic DSS also modestly elevated responses to 0.3 M MSG and upregulated expression of the *Tas1r1* subunit that transduces umami stimuli in taste cells*.* While we did not perform behavioral taste testing, we previously showed that milder, chronic gut inflammation suppressed brief-access licking and 23-h licking in addition to CT responses to sweet stimuli^24^. Thus, reduced neural responsiveness could potentially impair behavioral sensitivity to sweet tastants.

Several studies indicate that sweet taste may also be dysregulated in IBD patients. Patients with Crohn’s disease exhibit higher taste thresholds for sucrose compared to control subjects, which may lead to overconsumption of sugar^9, 41^. In other studies, however, IBD patients exhibited lower sensitivity to multiple taste modalities^8^, reduced sensitivity to bitter, salty, umami and fat but increased sour sensitivity^11^, or reduced sensitivity to multiple modalities except sour^7^. Others report no taste changes in patients^13^. Studies on IBD-induced taste dysfunction may lack consensus because of the inclusion of patients with both Crohn’s disease and ulcerative colitis, patients in different stages of flare-up and remission, varied dietary and environmental conditions, sex differences and the use of different methods to test taste sensitivity^1, 2, 7–11, 13^. In the current chronic study, we used male mice because acute disease symptoms were more pronounced, likely due to more robust inflammatory responses and protective effects of estrogen in the DSS model^31, 42^. Additional studies will determine whether female mice with colitis exhibit similar changes in sweet and umami taste function. Nonetheless, these results highlight the value of DSS-induced colitis in revealing mechanisms underlying taste changes in animals with defined environmental conditions and genetic backgrounds.

In addition to DSS, dietary and gut manipulations can alter peripheral taste function in rodents. Acute or chronic oral LPS gavage increased neutrophils in the colon, as shown here, and suppressed neural and behavioral responses to sweet stimuli^24, 25^. LPS also reduced CT nerve and behavioral responses to NaCl, but treated mice did not exhibit weight loss or sickness behavior unlike DSS suggesting divergent mechanisms^24^. Dietary manipulations with and without changes in body weight can also affect taste. Rats chronically consuming 30% sucrose solution did not gain significantly more weight compared to groups fed solid high-sucrose or control food, but CT nerve taste responses to sweet and salty stimuli decreased^39^. Fewer taste receptor cells responded to sweet stimuli and behavioral detection of sucrose and aceK was impaired in obese mice fed a high-fat diet ^43^. The same investigators showed that a high-fat diet, with and without weight gain, reduced the expression of gustducin and PLCβ2 downstream from sweet, umami and bitter receptor signaling as a mechanism for dampened taste responses^44^. The regulation of downstream taste transduction molecules in colitis, in addition to the decrease in sweet subunit expression identified here, will be of interest in future studies.

Mechanisms by which the inflamed colon affect taste buds remain to be determined. DSS is unlikely to damage taste buds directly since the lingual tissue structure is intact. The taste cell marker gene, K8, was also expressed at similar levels in DSS and control groups, indicating that taste buds do not undergo significant degeneration in mice with colitis. Though inflammation can regulate taste function^45, 46^, we found no evidence of increased immune cell responses near taste buds in DSS-treated vs. control mice. IBD can cause zinc deficiency^47, 48^, which impairs behavioral and neural taste responsiveness in rodents^49–51^. In previous studies, however, zinc deficiency suppressed CT responses to multiple taste qualities (i.e. NaCl, QHCl, HCl, NH_4_Cl, and glutamic acid)^50^. We did not measure serum zinc levels here, but found more restricted changes in neural responses to sweet stimuli and MSG. Malnourishment is a common symptom of IBD, and DSS-treated mice lost weight in the current study. Yet, CT responses to sodium were specifically impaired in rats maintained on a protein-deficient diet prenatally through adulthood^52^. Thus, there are mismatches in the taste modalities affected by dietary zinc and protein deficiency compared to mice with colitis.

DSS-induced colitis alters cytokine levels in serum as well as colon, providing a potential route of communication between the inflamed gut and taste buds. Circulating Th1-Th17 responses (i.e. TNF-α, IL-6, IL-17, CXCL-1) dominate in the acute phase after DSS treatment, while Th2 cytokines (i.e. IL-4 and IL-10) are chronically upregulated in mice with colitis^53, 54^. Oral taste bud cells express receptors for cytokines including TNF, IL-1, IL-6, and IL-10, which can also affect their function and life span^55–60^. Yet, the specific changes in sweet and umami taste observed in mice with chronic colitis limit potential upstream mechanisms. The Th2 cytokine, IL-10, is important for mucosal homeostasis^61^, and IL-10Rα (IL-10R1), the ligand-binding subunit of the IL-10 receptor, is co-localized to T1R3 + sweet- and umami-sensing taste cells^56^. However, serum IL-10 levels are elevated in mice with chronic DSS-induced colitis, while taste bud number and size decreased in the absence of IL-10^53, 54, 56^. The microbiota of the tongue, saliva and the GI tract is dysregulated in mice with colitis and patients with IBD^62–64^, which also could alter taste function. Type II taste cells express a genetic signature and possibly functions similar to microfold (M) cells that surveil mucosal-associated lymphoid tissues (MALT)^65^. Mice deficient in *Spib*, a transcription factor critical for M cell development, displayed increased licking responses to sucrose and MPG^65, 66^. While the parallels are imperfect, since neural responses to sweet and umami stimuli were inversely altered here, taste changes in colitis could depend on inflammatory or microbial dysregulation^66, 67^.

In conclusion, we report novel taste changes in male mice with chronic colitis. *Tas1r2* expression is suppressed and *Tas1r1* elevated in taste buds, mediating decreased sweet and heightened umami neural inputs to the brain. Chemosensory cells and receptor proteins in the gut and airway act as sentinels for microbes and coordinate inflammatory and neural responses^20, 68–71^. These results, together with previous studies^24, 25^, indicate that taste buds are also modulated by gut inflammation, which may in turn affect diet and nutrition. Changes in taste function could lead to excess consumption of palatable foods or nutritional deficits further exacerbating IBD, warranting further investigation^72–75^.

## Materials and methods

### Animals and DSS treatment

C57BL/6 J mice (#000664, Jackson Laboratory) were 9–10 weeks old at the beginning of treatment (day 0) after acclimatizing to the vivarium for at least 6 days. Mice were housed in a barrier facility with lights on a 12:12 light: dark cycle. All groups had free access to rodent chow (#2918; Envigo Teklad) throughout the study and filtered tap water between treatment periods. Mice were provided with 2% DSS (#160,110, 36–50 KDa, MP Biomedicals) dissolved in distilled water or distilled water alone in 15 ml tubes fitted with lixit valves. Mice were randomly assigned to the DSS or water control groups. We focused chronic studies on males since their weight loss was greater in preliminary acute studies (Fig. S1). Mice were weighed, fluid intakes recorded to confirm DSS ingestion, and stool samples collected daily during treatment and every 1–3 days between treatment periods. Sample sizes were based on previous work on gut-taste interactions, and are provided in figure legends^24, 25^. Animal procedures followed National Institutes of Health guidelines and were approved by the Augusta University Institutional Animal Care and Use Committee. Mice losing more than 25% of their original body weight were removed from the study and euthanized (*n* = 2) in compliance with a priori humane endpoints in our approved animal use protocol.

The effectiveness of DSS treatment in inducing colitis was verified by the disease activity index score (Fig. 1a), composed of summed grades for % body weight loss (Fig. 1b), blood in stool and stool consistency^34^. Body weight loss was scored as 0 (no loss), 1 (1–5%), 2 (> 5- 10%), 3 (> 10–20%) and 4 (> 20%). Hemoccult tests (Hemoccult Sensa; Beckman Coulter) were used to test for blood in stool samples which were graded as 0 (no blood), 1 (Hemoccult positive), 2 (Hemoccult positive and visual pellet bleeding) or 4 (gross bleeding and blood around anus). Stool consistency was graded as 0 (normal), 2 (loose stool), or 4 (diarrhea)^34^.

### Neurophysiology

We recorded CT nerve responses to tastants at day 56–61 following the first treatment period at day 0. Mice were anesthetized with ketamine (80 mg/kg) plus xylazine (10 mg/kg) i.p. and body temperature maintained at 36–39 °C. We injected supplemental anesthetic as needed. We tracheotomized mice, approached the CT nerve laterally and dissected it free of surrounding tissues, then placed it on a platinum electrode^24, 58^. Neural activity was amplified (Grass Instruments), integrated with a time constant of 1.5 s, and the summated signal recorded with PowerLab hardware and software (AD Instruments). Taste stimuli, applied to the tongue at room temperature with a syringe, included 0.10 and 0.50 M NaCl; monosodium glutamate (MSG; 0.3 M); 0.1, 0.5 and 1.0 M sucrose; 0.05 and 0.10 M saccharin; 0.025 and 0.05 M acesulfame (AceK); 1.0 M glucose; 16% polycose; 0.01 M quinine (QHCl), and 0.01N HCl mixed in distilled water. Chemical stimuli were purchased from Sigma with the exception of polycose (#00,746, Abbott Labs). CT nerve response magnitudes were calculated by subtracting the baseline from the height of the summated, integrated responses 20 s after stimulus application^24^. We normalized relative responses to mean 0.5 M NH_4_Cl responses recorded at the beginning and end of the stimulus series, and only analyzed bracketed series if 0.5 M NH_4_Cl responses deviated by ≤ 10%. At the end of recordings anesthetized mice were overdosed with isofluorane without awakening, followed by bilateral thoracotomy or cervical dislocation, which was also the method of euthanasia for all experiments below unless otherwise noted. Euthanasia methods comply with American Veterinary Medical Association (AVMA) standards.

### Immunofluorescence, histology and image analysis

Mice were euthanized as above and tongues removed on the mandible and fixed in paraformaldehyde for 5–7 h, then dissected free of the mandible, bisected longitudinally and cryoprotected overnight in 30% sucrose in PBS^58^. We used half of the tongue for qPCR (below) and half for immunostaining. Proximal and distal colons were dissected, flushed in PBS and mounted using the Swiss-roll technique^76^. Tongues and colons were flash-frozen in in O.C.T. (Sakura) and serially cryosectioned at 8 µm for immunostaining. Colon sections were incubated overnight at 4 °C in anti-mouse myeloperoxidase (MPO; 1:100; #AF3667, R&D) to identify neutrophils ^24^ followed by Alexa Fluor secondary antibody for 1 h at room temperature (1:1000; Jackson Immunoresearch). Nuclei were stained with DAPI (#D1306; Invitrogen / Thermo Fisher Scientific) and slides coverslipped with Fluoromount-G (#0100–01; SouthernBiotech). We identified macrophages in anterior tongue sections with CD68 (1:200; ab53444, Abcam) using similar procedures^58^. Sections of colon and tongue were also cleared and stained with hematoxylin and eosin.

MPO + neutrophils were analyzed in four distinct sections of colon using a microscope equipped with epifluorescence (Olympus BX51), a digital monochrome camera (Cool Snap, Roper Scientic) and MetaMorph software (MDS Analytical Technologies)^24^. A region of interest (12.9 mm^2^) was placed in the area of greatest MPO + cellular density within a section of colon and the image captured. MPO staining was thresholded, the % positive pixels / region calculated for each section of colon and the four values summed for each animal (Metamorph Software, Universal Imaging)^24^. The investigator was blinded to DSS or water treatment. We also captured brightfield images of hematoxylin and eosin-stained distal colon to assess inflammation and tissue breakdown. Disease scores for colons were assigned by an investigator blinded to treatment: colon architecture (normal, 0—severe crypt distortion with loss of crypts, 3); inflammatory infiltration (normal, 0—dense inflammation, 3); muscle thickening (normal, 0—marked muscle thickening, 3); goblet cell depletion (absent, 0—present, 1) and crypt abscess (absent, 0—present, 1). The individual scores were summed to determine the disease score for each animal^32, 34^.

DSS induces colitis by breaking down the gastrointestinal barrier^14^. While DSS is only briefly present in the oral cavity, we stained sections of tongue with hematoxylin and eosin to assess epithelial integrity and lingual inflammation using methods described above. We also analyzed CD68 + macrophages (1:100; Abcam) in fungiform papillae and the dorsal mucosa of the anterior two-thirds of the tongue^58^. Images were captured from four non-overlapping, standard-sized regions of interest (12.9 mm^2^) in the lingual epithelium and lamina propria, and four regions from the dorsal lingual mucosa. Image montages were assembled for each region, and CD68 + pixels thresholded to calculate the percentage of staining per standard area.

### Quantitative real-time polymerase chain reaction (qPCR)

We performed qPCR using previously described methods^58^. Tongue halves were stored in RNAlater (Fisher Scientific) at -80 °C until use. Primer sequences (Integrated DNA Technologies) are listed in Table 2. We performed qPCR using the QuantiTect SYBR Green PCR kit (Applied Biosystems) and the QuantStudio III Real-Time PCR Systems (Applied Biosystems). We normalized transcripts to *gapdh1* and used the 2^–ΔΔCT^ method to analyze changes in mRNA expression in DSS-treated relative to water-treated tongues^77^.

**Table 2:** Primers.

Gene accession #		Sequence 5’-3’	Size (bp)
*Tas1r1* (NM_031867.2)	Forward primer Reverse primer	TGGCAGCTATGGTGACTACG CAGCACCACAGACCTGAAGA	226
*Tas1r2* (NM_031873.1)	Forward primer Reverse primer	GCACCAAGCAAATCGTCTATCC ATTGCTAATGTAGGTCAGCCTCGTC	212
*Tas1r3* (NM_031872.2)	Forward primer Reverse primer	ACTACATACTGGGCGGGCTA GGTGAGAACCTGTTGCACGG	100
*Krt8* (NM_031170.2)	Forward primer Reverse primer	TCTTCTGATGTCGTGTCCAAGTG GATCCTCGGACGGGTCTCTAG	130
*Gapdh* (NM_001289726.1)	Forward primer Reverse primer	GGAAGGGCTCATGACCACAG TCACGCCACAGCTTTCCAG	81

### Statistical analyses

Statistical analyses were performed with Prism 9.4.1 (GraphPad Software). Colon disease scores, the density of MPO + neutrophils in colon, mRNA expression in lingual epithelium, and the density of lingual CD68 + macrophages were compared between DSS-treated and control groups with two-tailed Student’s t tests. One outlier was identified by Grubb’s test (α = 0.05; *G* = 2.488) in the *K8* mRNA data and not included in further analyses. To account for the repeated measurements on the same animal, we analyzed group differences in % body weight loss and disease index scores over time with linear mixed models fitted using R package lme4 (version 1.1–29). For comparing % body weight loss, *p* values were calculated using t tests with the degrees of freedom approximated by Satterthwaite’s method; for comparing disease index scores, *p* values were calculated using Wilcoxon rank-sum test. Neurophysiological responses were analyzed with two-way ANOVAs with treatment group and stimulus concentration as factors followed by Bonferroni post-tests^58^. We used Student’s t tests to analyze neural responses when we only one stimulus concentration was tested. We considered P values ≤ 0.05 significant in all statistical analyses.

### Supplementary Information

Supplementary Information.

## Data Availability

The datasets generated during and/or analyzed during the current study are available from the corresponding author upon reasonable request.
