# Supplementary material for: Altered peripheral taste function in a mouse model of inflammatory bowel disease
Source: Sci Rep. 2023 Nov 2;13:18895. doi: 10.1038/s41598-023-46244-3 (PMC10622515; doi:10.1038/s41598-023-46244-3)
Supplement: Supplementary file 1 — Supplementary Information. [file 41598_2023_46244_MOESM1_ESM.pdf]

Supplementary results

Altered peripheral taste function in a mouse model of inflammatory bowel disease

Authors: Guangkuo Dong[1], Schuyler Kogan[1], Khaylie Boothe[1], Lianying He[1], Yang Shi[2] and *Lynnette Phillips McCluskey[1]

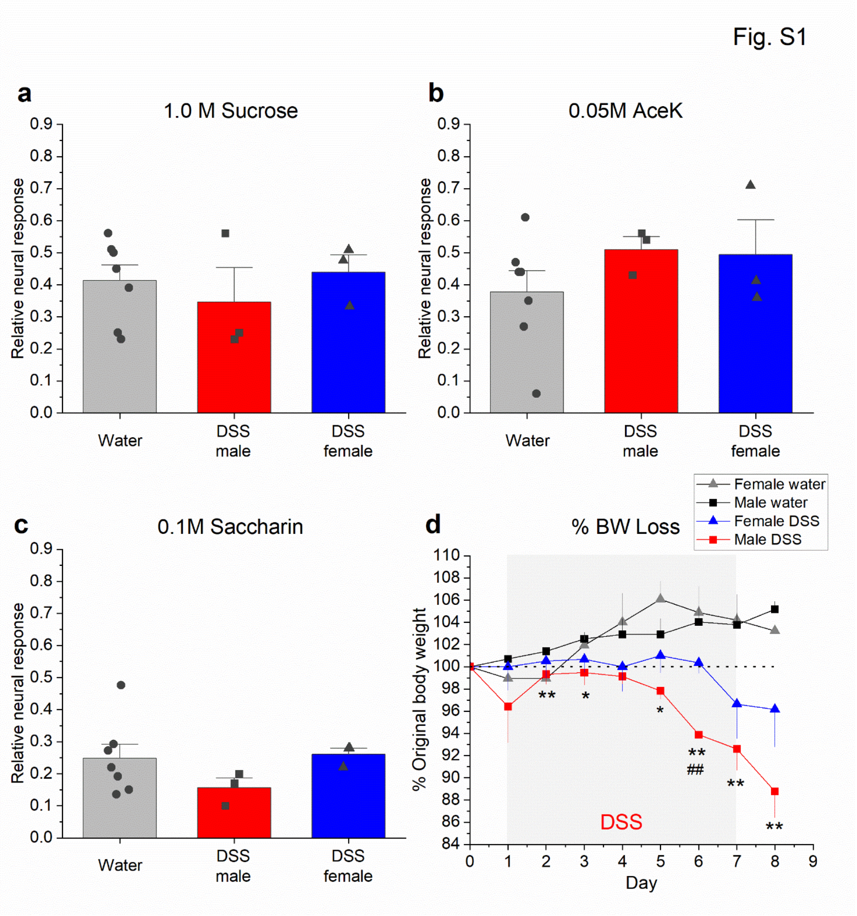

<u>Figure S1</u>. Acute DSS did not alter neural taste responses though males lost body weight. Male and female mice were provided with 3% DSS in drinking water (n=3 each sex) or water (n=3-4 each sex) alone for 7 days. CT responses to taste stimuli were recorded 21-25 days later. Responses to **a** sucrose, **b** aceK, **c** saccharin, (not shown) NaCl, polycose, glucose, HCl or QHCl were similar between groups. Neural responses were comparable in male and female groups maintained on water ($p > 0.05$), and are collapsed in panels **a-c**. **d** There were no significant differences in body weight in male vs. female mice maintained on water alone ($p > 0.05$) or in female water vs. female DSS-treated groups ($p > 0.05$) from day 1-8. Males receiving DSS lost significantly more body weight than male controls on day 2, 3, and 5-8.

Actual *p* values are shown in Table S1. DSS-treated males also lost significantly more of their original body weight compared to DSS-treated females at day 6 ($p = 0.003$). *$p < 0.05$; **$p < 0.01$ in male treated vs. control; ## $p < 0.01$ DSS male vs. DSS female.

TABLE S1
Statistics for % body weight loss after acute DSS treatment: Male Water vs. Male DSS

Day[a]	*t* statistic	*p* value
0	N/A	N/A
1	-2.10	0.103
2	-5.25	0.006**
3	-3.96	0.017*
4	-2.51	0.066
5	-3.15	0.034*
6	-6.70	0.003**
7	-7.21	0.002**
8	-7.93	0.001**

a. Mice treated with DSS on shaded days. b. N/A, all values 100%
